# miR-28-5p and miR-708-5p Share a Common Seed with Different Functions in Lung Cancer Patients

**DOI:** 10.3390/ijms262110364

**Published:** 2025-10-24

**Authors:** Cristina Alexandra Ciocan, Cecilia Bica, Liviuta Budisan, Lajos Raduly, Sergiu Chira, Claudia-Cristina Burz, Ovidiu Farc, Antonia Harangus, Marioara Simon, Constantin-Ioan Busuioc, Stefan Strilciuc, Cornelia Braicu, Ioana Berindan-Neagoe

**Affiliations:** 1 Department of Genomics, MEDFUTURE Institute for Biomedical Research, Iuliu Hațieganu University of Medicine and Pharmacy, 400337 Cluj-Napoca, Romania; crisciocan@gmail.com (C.A.C.); cecilia.bica8@gmail.com (C.B.); liviuta.budisan@umfcluj.ro (L.B.); lajos.raduly@umfcluj.ro (L.R.); sergiu.chira@umfcluj.ro (S.C.); farc.ovidiu@yahoo.com (O.F.); antonia.harangus@yahoo.com (A.H.); strilciuc.stefan@umfcluj.ro (S.S.); ioana.neagoe@umfcluj.ro (I.B.-N.); 2 Department of Immunology and Allergology, Faculty of Medicine, Iuliu Hatieganu University of Medicine and Pharmacy, 400337 Cluj-Napoca, Romania; cburz@yahoo.fr; 3 Department of Medical Oncology, The Oncology Institute “Prof. Dr Ion Chiricuţă”, 400015 Cluj-Napoca, Romania; 4 Leon Daniello Pulmonology Hospital, 400332 Cluj-Napoca, Romania; simonmariaro@gmail.com; 5 Department of Pathology, Clinical Hospital Sfanta Maria, Bulevardul Ion Mihalache 37-39, 011172 București, Romania; 6 Department of Pathology, Onco Team Diagnostic, 010719 Bucharest, Romania; 7 Doctoral School, Iuliu Hatieganu University of Medicine and Pharmacy, 400337 Cluj-Napoca, Romania; 8 Academy of Medical Sciences, 020021 Bucharest, Romania

**Keywords:** lung cancer, biomarker, miR-28-5p, miR-708-5p, miRNA seed sequence

## Abstract

Lung cancer remains the leading cause of cancer-related mortality worldwide, accounting for nearly 1.8 million deaths annually. The present study aimed to investigate the role of miR-28-5p and miR-708-5p in lung cancer and to analyze the relationship between target gene profiles and transcriptional factor regulation. Both miRNAs that share a common seed sequence were found to be overexpressed in a cohort of 32 paired tumor and adjacent normal tissue samples collected from patients diagnosed at advanced stages (III and IV) of disease. Data from the dbDEMC database revealed that miR-28-5p exhibited variable expression across lung cancer subtypes, whereas miR-708-5p showed consistent overexpression, reinforcing its potential clinical diagnostic significance. Using the TransmiR database, we identified complex TF–miRNA regulatory networks, with both shared and distinct transcription factors controlling miR-28-5p and miR-708-5p. Pathway enrichment analysis indicated that these miRNAs regulate several cancer-associated pathways, including ECM–receptor interaction, adherens junctions, and Hippo signaling. Overall, our findings suggest that miR-708-5p may have a potential clinical application in lung cancer.

## 1. Introduction

Lung cancer includes three major histological subtypes: non-small cell lung cancer (NSCLC), which accounts for approximately 80–85% of cases, small cell lung cancer (SCLC), representing about 10–15%, and malignant pleural mesothelioma (MPM), a rare subtype contributing to less than 1% of primary lung malignancies. Meanwhile, the NSCLC was subclassified as lung adenocarcinoma (LUAD) and lung squamous cell carcinoma (LUSC). This condition is the leading cause of cancer-related deaths, with an estimated 1.8 million deaths due to late diagnosis [1]. Therefore, the mortality rate is very high, and the 5-year overall survival rate is only 15% [2]. Identifying novel molecular candidates with clinical utility to understand better this malignancy remains a key objective in clinical research and development. The pathogenesis of lung cancer is complex, involving both genetic and transcriptomic alterations [3].

MiRNAs are small non-coding RNAs, 19–24 nucleotides in length, that serve as critical regulators of gene expression through a post-transcriptional mechanism, thereby impacting various physiological and pathological cellular processes [4,5,6,7]. Mounting evidence has shown that miRNAs play various regulatory roles, depending on the target genes, acting as oncogenes or tumor suppressors [6,7,8,9,10]. Significant alterations of miRNA expression patterns in tumor tissue versus normal adjacent tissue make them potential molecule candidates with clinical utility [7,11]. The precise contribution of individual miRNAs remains incompletely understood [3,12,13,14,15,16,17], including in lung cancer [3,15,16,17].

miR-28 and miR-708 are key transcripts with a common seed sequence implicated in cancer biology [18,19]. MiR-28-5p has been proven to have emerging roles in cancers targeting critical pivotal factors related to cell proliferation, migration, invasion, and metastasis [20,21,22]. Similarly, miR-708-5p is dysregulated in various cancers, where it can be either overexpressed or underexpressed compared to normal adjacent tissue [23,24]. Therefore, miR-708-5p appears to have both tumor-suppressor and tumor-promoting functions [23,25].

This study investigated the roles of miR-28-5p and miR-708-5p in lung cancer, with a specific focus on their potential clinical applications. The novelty of this work lies in the comparative analysis of two miRNAs with same seed sequence, highlighting their differential expression patterns in lung cancer and the regulatory networks they influence. We evaluated their expression in paired NSCLC tumor and adjacent normal tissues and validated our findings with additional data from the dbDEMC database. Furthermore, we identified target genes, regulatory transcription factors networks, and miRNA-gene associated networks, highlighting signaling pathways regulated by these transcripts.

## 2. Results

### 2.1. miR-28-5p and miR-708-5p Share a Common Seed Sequence

**Figure 1:**
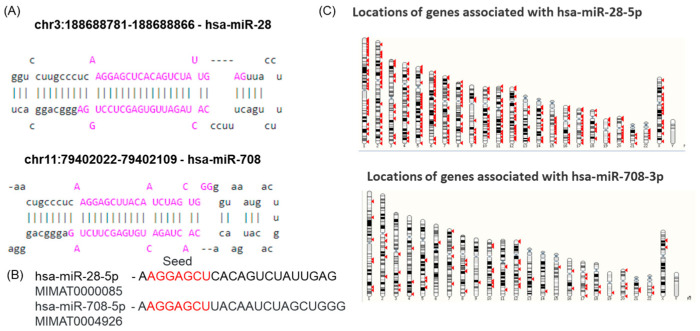
Chromosomal location, structure, and sequence of miR-28 and miR-708. (**A**) Chromosomal positions of pre-miR-28 and pre-miR-708 and their structures; the genomic localisation of the miR-28-5p and miR-708-5p family members was determined using the UCSC genome browser (https://genome.ucsc.edu) and miRBase; (**B**) hsa-miR-708-5p and hsa-miR-28-5p sequences containing the seed region (red) that is common to both transcripts, downloaded from miRBase (https://mirbase.org/hairpin/MI0000086, 24 August 2024); (**C**) chromosomal locations of genes related to miR-28 and miR-708, downloaded from the Ensembl Genome Browser (https://www.ensembl.org/, 24 August 2025).

Genomic mapping indicated that miR-28-5p is encoded on chromosome 3 within an intronic region of the LPP (LIM domain containing preferred translocation partner in lipoma) gene, whereas miR-708-5p is encoded on chromosome 11, in the intronic region of the gene TENM4 (teneurin transmembrane protein 4), as observed from the MIRIAD database. The stem-loop structures of these transcripts, downloaded from miRBase, are presented in Figure 1A.

The specific sequence region responsible for target recognition is called the “seed sequence”, which comprises 2–8 nucleotides at the 5′ end, as shown in Figure 1B for the two selected transcripts. Since miRNAs can regulate multiple target genes, their functional effectiveness depends not only on seed complementarity but also on interactions mediated by the non-seed region. Variations in this region can change target specificity and binding affinity, ultimately leading to different regulatory outcomes. Structural differences outside the seed region may also influence miRNA stability, accessibility, and interaction with mRNAs. Furthermore, the two miRNAs differ in both the number and chromosomal distribution of their predicted targets (Figure 1C), supporting the idea that, despite sharing a seed sequence, miR-28-5p and miR-708-5p likely have distinct biological roles.

**Figure 2:**
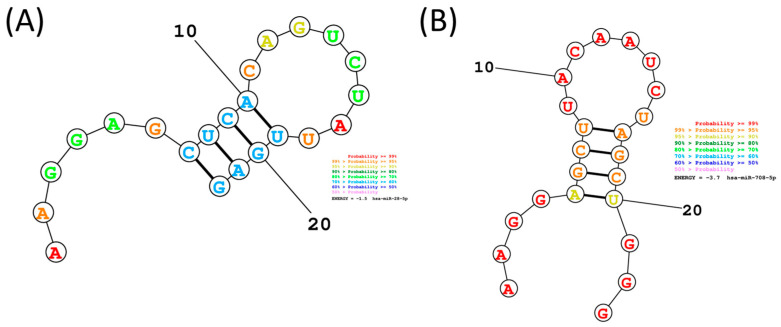
miR-28-5p and miR-708-5p secondary structure prediction. This is the predicted lowest free energy structure for the two-transcript sequence for the ‘Predict a Secondary Structure’ server of the RNAstructure (**A**) miR-28-5p and (**B**) miR-708-5p. It is color-annotated according to base-pairing probability.

The secondary structure prediction and analysis of miR-28-5p and miR-708-5p, using RNAstructure, provide insights into their thermodynamic stability, which is directly linked to their biological function. As can be observed from Figure 2, the free energy for miR-28-5p is −1.5, meanwhile for the case of miR-708-5p is −3.7; the secondary structure forms due to the folding of the sequence into paired and unpaired regions, contributing to its overall stability and potential interactions with target mRNAs, therefore miR-708-5p have a higher stability, it is expected that transcripts might have a context dependent functionality.

### 2.2. qRT-PCR for miR-28-5p and miR-708-5p Expression Assessment in NSCLC Patients

**Figure 3:**
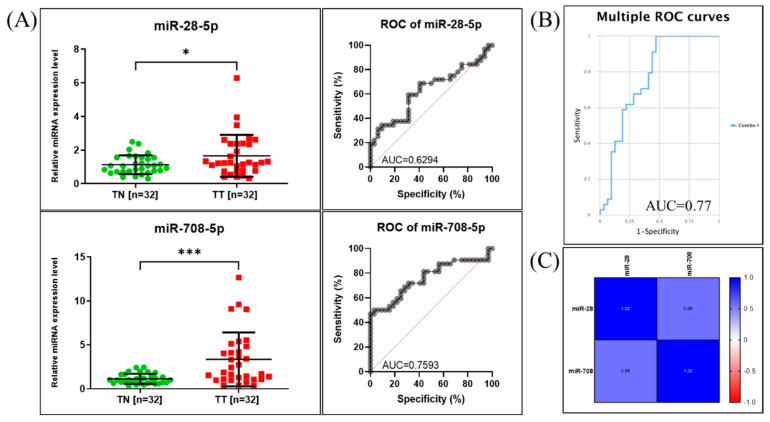
miR-28-5p and miR-708-5p in NSCLC patients. (**A**) Expression of miR-28-5p and miR-708-5p in TT vs. TN (* *p* ≤ 0.05, *** *p* ≤ 0.001) and ROC curve for these data; (**B**) Graphic representation of multiple ROC curve for markers in combination are analyzed using CombiROC online tool; (**C**) Pearson correlation among the expression levels of miR-28-5p and miR-708-5p.

When analyzing the relative expression levels of the tested miRNAs, we observed increased levels for both miRNAs when comparing tumor to adjacent non-tumor tissue samples (Figure 3). The relative expression in tumor tissue was 1.625 ± 1.245 (*p* = 0.0311) for miR-28-5p and 3.351 ± 3.066 (*p* = 0.0002) for miR-708-5p.

To further assess their clinical relevance, we performed ROC curve analysis, which evaluates each miRNA’s ability to differentiate between tumor and non-tumor samples. The area under the curve (AUC) provides a quantitative measure of diagnostic accuracy, with values closer to 1.0 indicating better discriminatory power and a value of 0.5 indicating no discriminative ability. The analysis revealed that miR-28-5p had an AUC of 0.6294, suggesting modest discriminatory capacity, while miR-708-5p achieved an AUC of 0.7593, indicating a higher ability to distinguish tumor from non-tumor tissues.

The combined ROC curve, generated using the CombiROC online tool [26], displayed an AUC value of 0.77 (Figure 3B), demonstrating slighly improved diagnostic performance when the two miRNAs were analyzed together compared to individual assessment. Figure 3C shows the statistical correlation between the expressions of the two evaluated miRNAs.

### 2.3. Validation of the Expression Levels of miR-28-5p and miR-708-5p in Lung Cancer Using Public Available Datasets

The expression profiles of miR-28-5p and miR-708-5p in lung cancer, obtained from the dbDEMC database, are presented in Table 1 and Figure S1, supporting validation of laboratory data through external datasets [27]. The expression of miR-28-5p shows some variation across different lung cancer subtypes, suggesting potential context-dependent roles. In contrast, the expression of miR-708-5p appears consistent across lung cancer datasets, indicating a more uniform regulatory pattern.

### 2.4. Functional Classification and Enrichment Analysis of miR-28-5p and miR-708-5p Target Genes

**Figure 4:**
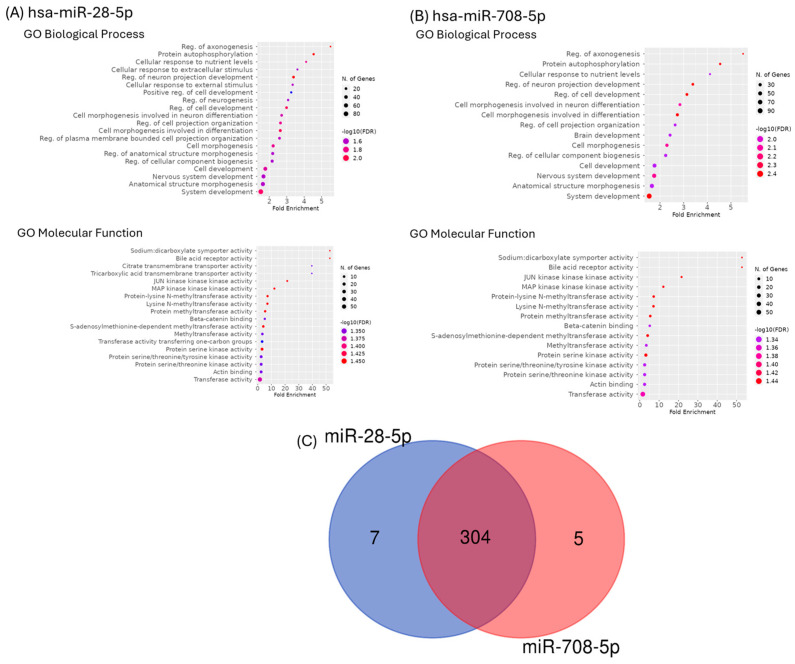
Gene Ontology (GO) term enrichment analysis for (**A**) miR-28-5p and (**B**) miR-708-5p, emphasizes main biological processes and molecular function (**C**) Venn diagram emphasizes common targets for the two transcripts. An FDR of <0.05 was used to pick significantly enriched GO terms.

GO enrichment approaches were used to identify the putative roles of the main targets for the selected two transcripts (Figure 4A,B). GO enriched categories in biological process groups were primarily linked to system development, anatomical structure morphogenesis, or nervous system development. GO enriched categories in molecular function were mainly related to transferase activity, actin binding, or protein serine/threonine kinase activity. A diagram Venn was generated, and we can observe 304 common genes (Figure 4C).

### 2.5. Pathway Analysis for miR-28-5p and miR-708-5p

**Figure 5:**
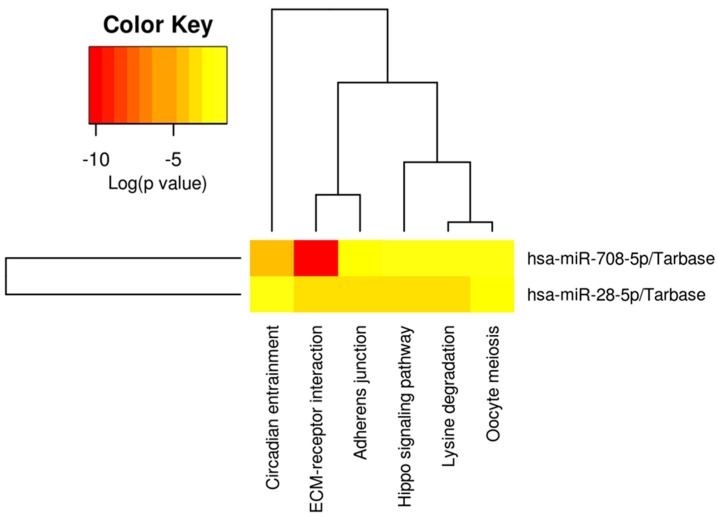
Pathway and target analysis of miR-28-5p and miR-708-5p. Heatmap showing KEGG pathway enrichment of miR-28 and miR-708.

The pathway and target analysis of miR-28-5p and miR-708-5p was performed using DIANA Tools mirPath v.3 (Table 2). In addition, Figure 5 represents the heatmap showing the pathway analysis for the two transcripts, revealing ECM–receptor interaction (Figure S2), adherence junctions (Figure S3), and Hippo signaling (Figure S4) as key elements.

While these analyses provide a comprehensive in silico overview of target gene networks and signaling pathways, we acknowledge that they remain predictive in nature. Experimental validation of target gene expression and pathway activity will be essential to confirm the functional impact of these findings. Nonetheless, this computational framework identifies clinically relevant candidates and prioritizes ECM–receptor interactions, adhesion molecules, and Hippo signaling as key mechanisms for future translational studies in lung cancer.

### 2.6. Transcription Factors and Regulatory Networks

**Figure 6:**
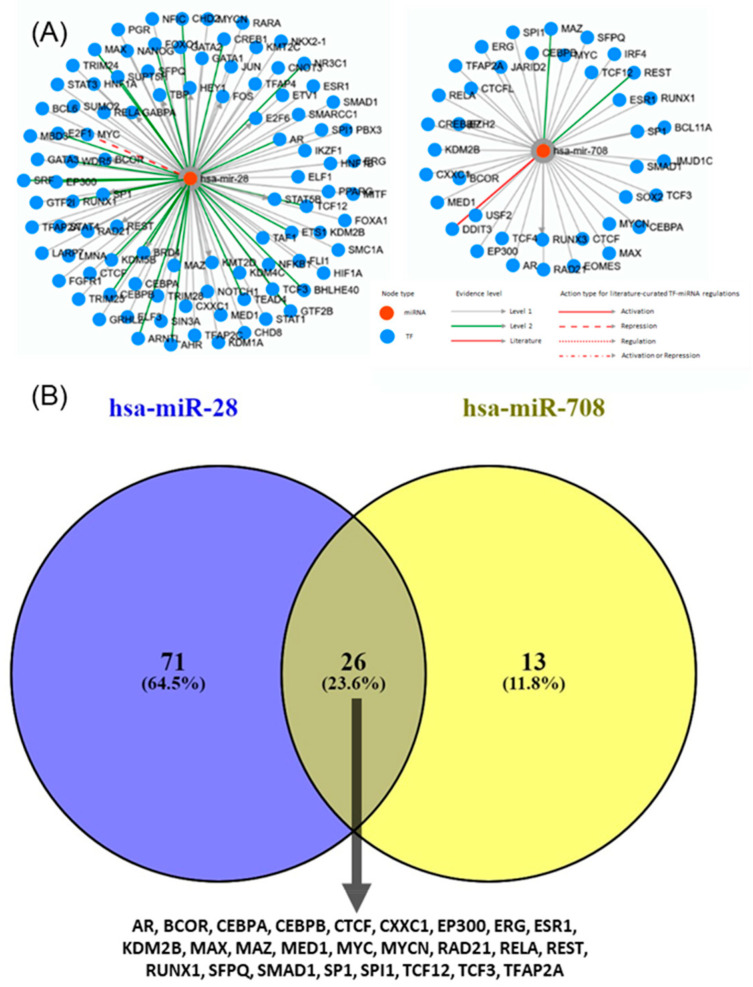
TF and regulatory networks for miR-28 and miR-708. (**A**) Relationship of miR-28 and miR-708 with TF and regulatory networks (TransmiR v2.0, http://www.cuilab.cn/transmir, accessed on 22 October 2025); (**B**) Venn diagram indicating the common and specific TF for the two transcripts.

TransmiR is a database that provides regulatory relationships between transcription factors (TFs) and miRNAs. Predicting the regulation of miR-28-5p and miR-708-5p based on TF binding motifs revealed complex interactions embedded within broader transcriptional networks involving multiple TFs. Both miRNAs were linked to extensive TF regulatory networks (Figure 6A). Although a subset of 26 TFs was shared, each miRNA also displayed unique TF associations, reflecting distinct layers of transcriptional control (Figure 6B). Interestingly, despite possessing a common seed sequence, these miRNAs appear to be regulated by different TFs and may therefore contribute to divergent biological roles in cancer, underscoring the complexity of their transcriptional regulation.

## 3. Discussion

Lung cancer patients are diagnosed in late stages, when the treatment strategies are related to metastatic disease, and the patients usually develop drug resistance [11,28,29,30,31]. In this study, we examined miR-28-5p and miR-708-5p, whose overexpression suggests potential oncogenic roles in lung cancer progression. Both miRNAs were significantly upregulated in tumor tissues compared with adjacent normal tissues (Figure 3), with further validation in the dbDEMC database. Notably, miR-28-5p displayed subtype-specific expression variability, indicating a possible role in tumor heterogeneity, whereas miR-708-5p showed consistent overexpression. ROC curve analysis reinforced these findings, as miR-708-5p demonstrated stronger discriminative ability between tumor and non-tumor tissues, underscoring its potential clinical utility in diagnosis.

MiR-28-5p expression is upregulated in NSCLC tumor tissues and cell lines [8,22]. In a recent study, the miR-28/PTEN axis is a crucial pathway that may serve as a potential clinical target for diagnosis, treatment, and prognosis [8]. Another study demonstrated that miR-28-5p can promote cancer progression by regulating the level of HIF-1α [22].

MiR-708-5p was identified as overexpressed in several lung cancer datasets [32], being associated with an increased risk of death after adjustments for all clinically significant factors, including age, sex, and tumor stage [33]. Overexpression of miR-708 in tumors is also related to poor overall survival, particularly in never-smoking lung adenocarcinoma patients [33]. Moreover, miR-708-5p has been associated with WNT regulation [33], β-catenin signaling [34], EMT, drug resistance and metastasis [35]. To our knowledge, no direct study that presents interaction with genes related to ECM, adhesion molecules, or Hippo signaling has been published. The oncogenic effects of miR-708 could be attributed to its interactions with specific target genes and pathways that regulate cell proliferation, survival, and metastasis [23,33,34,36]. The present study identified a significant number of genes targeted by the transcripts mentioned above, which appear to be involved in ECM–receptor interaction, including five that are correlated with the overall survival rate of NSCLC patients. A deeper comprehension of the structural and functional role of the ECM in NSCLC progression can be used to identify novel potential therapeutic targets, particularly in the case of high-risk groups [37]. These genes are known to be involved in cancer cell invasion [37,38].

Adherens junction proteins act as tumor suppressors or promoters of cell transformation [39]. The alteration of this class of genes has been widely reported in several solid tumors [40], which might have a different impact depending on the cancer type and stage [40]. Several of these genes were proved to be related to the differential infiltration of immune cells in the NSCLC [41].

MiRNAs were connected to the Hippo pathway in tumor progression [42], this finding is also supported by the present study, which highlights several target genes involved in this pathway; importantly, a substantial proportion of these genes show a correlation with overall survival. No direct information related to the connection of miR-28 and miR-708 and Hippo signaling was previously reported, even though the Hippo pathway has been reported to be closely associated with lung cancer [42].

A limitation of the present study is that our analysis related to target genes is confined to in silico assessments. Therefore, the clinical significance of our data has yet to be evaluated. Additional research is needed to better understand the system governing ECM, adherens, or Hippo signaling. Further studies, including functional experiments, mechanistic investigations, and analysis on larger patient cohorts, are needed to comprehensively understand the roles of miR-28-5p and miR-708-5p overexpression in NSCLC and to determine their potential as therapeutic targets or diagnostic/prognostic markers.

MiR-28-5p and miR-708-5p share a common seed sequence. However, their distinct biological properties may arise from differences in their target genes and cellular contexts, as well as their genomic localization. The seed sequence of miRNA is vital for target recognition and binding. Nevertheless, other factors, including target site accessibility, secondary structure, and interactions with other molecules, also influence the miRNA’s regulatory effects [43]. The presence of a shared seed sequence supports partial functional redundancy at the post-transcriptional level. Simultaneously, differences in TF regulation imply they could be activated under different cellular or pathological conditions, adding flexibility to cancer-related gene regulatory networks. Currently, there are no experimentally verified entries linking specific TFs to these transcripts. This gap indicates that the upstream transcriptional regulation of miR-28-5p and miR-708-5p remains underexplored. Understanding which TFs regulate their expression in NSCLC may help explain their different behaviors despite their shared seed sequence. This duality, with shared targets yet distinct regulation, emphasizes the complexity of miRNA–TF networks and may explain why these TE-derived miRNAs exhibit both overlapping and unique roles in tumor biology.

## 4. Materials and Methods

### 4.1. Genomic Localisation and Sequence Analysis

The genomic loci of miR-28-5p and miR-708-5p were identified using the MIRIAD database (https://www.miriad-database.org/miRNA/human/hsa-mir-708, accessed 12 August 2025). Pre-miR and the mature sequences of miR-28-5p and miR-708-5p were retrieved from miRbase [44], and their seed sequences (nucleotides 2–7/8) were identified and highlighted to assess sequence conservation. Sequences for mature hsa-miR-28-5p (accession number MIMAT0000085) and hsa-miR-708-5p (accession number MIMAT0004926) were retrieved from miRBase (http://mirbase.org, accessed 12 August 2025) and used as input to generate the secondary structures by RNAstructure, version 6.5 software [45].

### 4.2. Patients

This study included 32 patients diagnosed with NSCLC according to internationally accepted criteria. All the patients included in the study signed informed consent. For each patient, we stored fresh frozen tumors (TT) and their paired adjacent non-tumor tissue (TN) in a biobank. Table 3 summarizes the clinicopathological data for the NSCLC cases used for the present study.

### 4.3. Evaluation of miR-28-5p and miR-708-5p by Quantitative Real-Time PCR (RT-PCR)

Fresh frozen tissue was used for RNA extraction using the classical TriReagent-based method, as previously described [4]. For the evaluation of expression levels of the two transcripts, TaqMan MicroRNA Transcription kit (ThermoFischer Scientific, Waltham, MA, USA) and TaqMan microRNA primer assay (ThermoFischer Scientific, Waltham, MA, USA) were used, in parallel with the evaluation of U6 and RNU48 as housekeeping miRNAs as previously described [4]. Table 4 presents the sequence of the primers of the tested miRNAs. The data was analyzed using the ΔΔCT method and graphical representation. Statistical analysis of the relative expression levels in TT versus TN was done using GraphPad Prism software v.9 (GraphPad Software, San Diego, CA, USA).

### 4.4. miR-28-5p and miR-708-5p Expression Levels in Lung Cancer Public Database

Expression data for these two transcripts for lung cancer were retrieved from dbDEMC (Database of Differentially Expressed miRNAs in Human Cancers) [27,46], a curated resource that integrates high-throughput expression profiles from microarray and RNA-seq studies. dbDEMC provides systematically annotated information on miRNA expression across multiple cancer types, enabling comparison between tumor and normal samples [27,46].

### 4.5. Statistical Analysis

The differences between TT and TN were calculated using the *t*-test, with a significance of *p* < 0.05. Correlation analysis was performed employing the Pearson correlation coefficient. All analyses were conducted using GraphPad Prism software v.9 (GraphPad Software, San Diego, CA, USA). Additional graphical representation of receiving operator characteristics (ROC curve) analysis for the individual transcripts was done using GraphPad Prism software v.9, and the combined ROC curve for the two transcripts was performed using the CombiROC online tool (http://combiroc.eu/) [26].

### 4.6. Predicted Target Genes Enrichment Analysis

Target genes prediction for hsa-miR-28-5p and hsa-miR-708-5p were made using miRDB webtool [47]. For each miRNA, a gene set enrichment analysis of the predicted target genes was performed using ShinyGO version 0.80 [48]. Charts for gene ontology (GO) biological processes and molecular functions were generated using a false discovery rate (FDR) cutoff limit of 0.05, and data was sorted by FDR on *X* axis.

### 4.7. Biological Pathways Associated with miR-28b-5p and miR-708-5p

Predictions of potential targets of these transcripts were performed by computational algorithms based on ‘seed regions’ between miRNAs and target genes using TargetScan, 8.0 (http://www.targetscan.org, 20 August 2024) [49] and TFs TransmiR v3.0 (Scilab.cn, 22 August 2024) was used [50].

DIANA-miRPath v3.0 (http://www.microrna.gr/miRPathv3, accessed on 22 August 2024), an online software suite, was used for the assessment of miRNA regulatory roles and the identification of pathways modulated by the two miRNAs [49,51]. We were able to identify the main pathways regulated by the selected two transcripts. The interactions between miR-28-5p and miR-708-5p, as well as their related target genes, were identified using the miRNet online tool [52].

## 5. Conclusions

Our study highlights the oncogenic potential of miR-28-5p and miR-708-5p in lung cancer, as both were found to be overexpressed in tumor tissues compared with normal tissues, a finding validated using dbDEMC. While the two miRNAs share a common seed sequence and a relatively high number of target genes, they appear to be regulated by different TF. Notably, miR-28-5p displayed subtype-specific variability as observed from data downloaded from dbDEMC, potentially contributing to tumor heterogeneity, whereas miR-708-5p showed consistent expression patterns and stronger diagnostic performance, reinforcing its clinical utility.

While our findings provide comparative insights into two sequence-related miRNAs with distinct roles in lung cancer biology and clinical diagnosis, the study is limited by its reliance on in silico analyses. Future experimental validation will be necessary to confirm the regulatory mechanisms and strengthen their clinical application.

## Figures and Tables

**Table 1:** Expression levels of miR-28-5p and miR-708-5p in lung cancer datasets obtained from dbDEMC.

miRNA ID	Source ID	Cancer Type	Design	logFC	AveExpr	*p* Value	adj *p* Value	Expression Level
hsa-miR-28-5p	GSE135918	lung cancer	cancer vs. normal	−0.74	0.44	0.000417	0.00745	DOWN
hsa-miR-28-5p	GSE74190	lung cancer	cancer vs. normal	0.55	4.91	0.000374	0.00304	UP
hsa-miR-28-5p	GSE74190	lung cancer	cancer vs. normal	0.93	4.91	0.0000000398	0.000000309	UP
hsa-miR-28-5p	TCGA_LUAD	LUAD	cancer vs. normal	0.25	6.68	0.00648	0.0163	UP
hsa-miR-28-5p	TCGA_LUSC	LUSC	cancer vs. normal	−0.2	6.89	0.00566	0.0287	DOWN
hsa-miR-708-5p	SRP040720	lung cancer	cancer vs. normal	1.83	5.79	0.000630	0.00726	UP
hsa-miR-708-5p	TCGA_LUAD	LUAD	cancer vs. normal	2.53	4.57	4.66 × 10^−29^	5.96 × 10^−28^	UP
hsa-miR-708-5p	TCGA_LUSC	LUSC	cancer vs. normal	3.24	6.47	2.34 × 10^−51^	2.47 × 10^−49^	UP

**Table 2:** Extrapolation of the main KEGG pathway based on the target genes related to miR-28-5p and miR-708-5p using the Diana Tools mirPath v.3 database.

	KEGG Pathway	*p*-Value	#Genes	#miRNAs	Target Genes
1	ECM–receptor interaction	4.46 × 10^−13^	8	2	**miR-28-5p:** AGRN, HSPG2, COL6A2, COL1A1, COL1A2, LAMC1
					**miR-708-5p:** AGRN, COL6A1, COL6A2, COL1A1, LAMC1
2	Adherents junction	1.44 × 10^−5^	12	2	**miR-28-5p:** ACTB, CTNND1, LMO7, IQGAP1, IGF1R, EGFR, SMAD4, CTNNB1, FARP2, MAPK1
					**miR-708-5p:** SMAD2, IQGAP1, IGF1R, CTNNB1, PVRL1
3	Lysine degradation	3.70 × 10^−5^	7	2	**miR-28-5p:** WHSC1L1, NSD1, ASH1L, KMT2D, KMT2A, KMT2B
					miR-708-5p: NSD1, WHSC1, KMT2A
4	Hippo signaling pathway	6.01 × 10^−5^	17	2	**miR-28-5p:** ACTB, YWHAH, YWHAE, BTRC, CCND2, CSNK1D, DLG4, CCND1, SMAD4, CTNNB1, TEAD1, STK3, PPP2CB, PPP2R1B, BMPR2
					**miR-708-5p**: YWHAH, SMAD2, YWHAE, CCND2, BIRC5, CTNNB1, STK3, BMPR2

**Table 3:** Clinico-pathological characteristics of the patients used for the tissue matched/paired samples qRT-PCR evaluation of miRNA expression.

Characteristics		No. of Patients (%)
Age (years)	≤65	16 (50)
	≥65	16 (50)
T	T2	4 (12.5)
	T3	9 (28.1)
	T4	19 (59.4)
N	N0	3 (9.4)
	N1	3 (9.4)
	N2	21 (65.6)
	N3	5 (15.6)
M	M0	18 (56.3)
	M1	14 (43.7)
Stage	III	19 (59.4)
	IV	13 (40.6)

**Table 4:** Sequences of tested miRNAs.

miRNA	Assay Code	miRNA Sequence
RNU48	0001006	GATGACCCCAGGTAACTCTGAGTGTGTCGCTGATGCCATCACCGCAGCGCTCTGACC
U6	001973	GTGCTCGCTTCGGCAGCACATATACTAAAATTGGAACGATACAGAGAAGATTAGCATGGCCCCTGCGCAAGGATGACACGCAAATTCGTGAAGCGTTCCATATTTT
miR-28-5p	000411	AAGGAGCUCACAGUCUAUUGAG
miR-708-5p	002341	AAGGAGCUUACAAUCUAGCUGGG

## Data Availability

The original contributions presented in this study are included in the article. Further inquiries can be directed to the corresponding authors.

## Supplementary Materials

The supporting information can be downloaded at https://www.mdpi.com/article/10.3390/ijms262110364/s1.
