# Supplementary material for: miR-28-5p and miR-708-5p Share a Common Seed with Different Functions in Lung Cancer Patients
Source: Int J Mol Sci. 2025 Oct 24;26(21):10364. doi: 10.3390/ijms262110364 (PMC12607647; doi:10.3390/ijms262110364)
Supplement: Supplementary file 1 [file ijms-26-10364-s001.zip › ijms-3923393-supplementary.pdf]

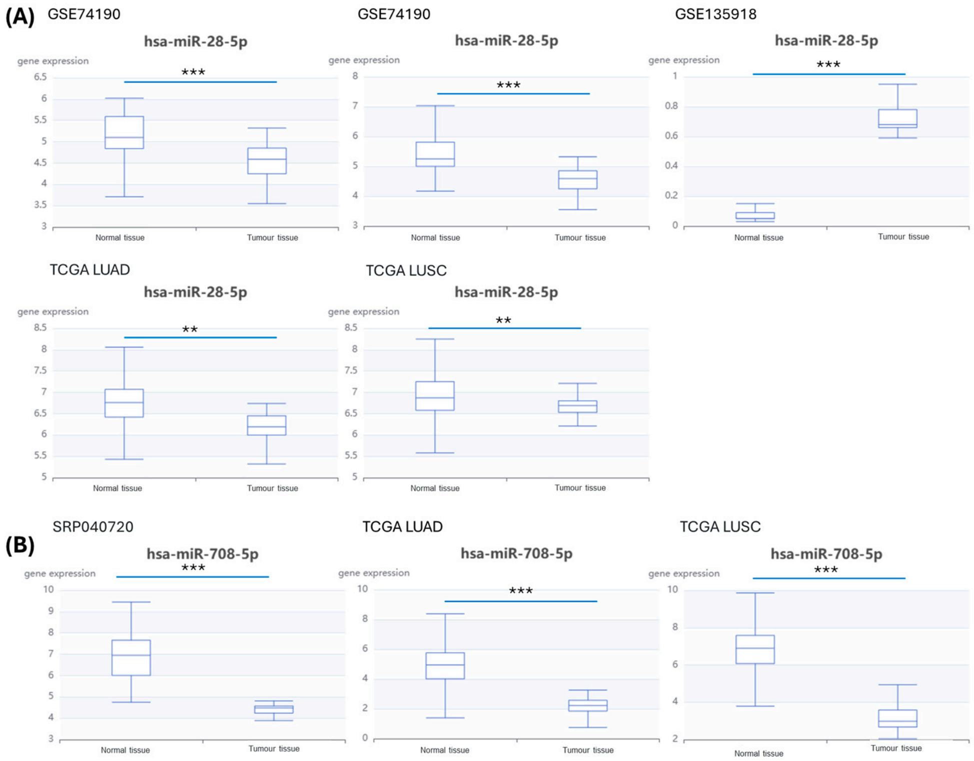

Figure S1. Graphical representation of the expression levels of miR-28-5p and miR-708-5p in lung cancer datasets obtained from dbDEMC.(A) miR-28-5p expression levels (B) miR-708-5p (p** ≤ 0.01, ***P ≤ 0.001)

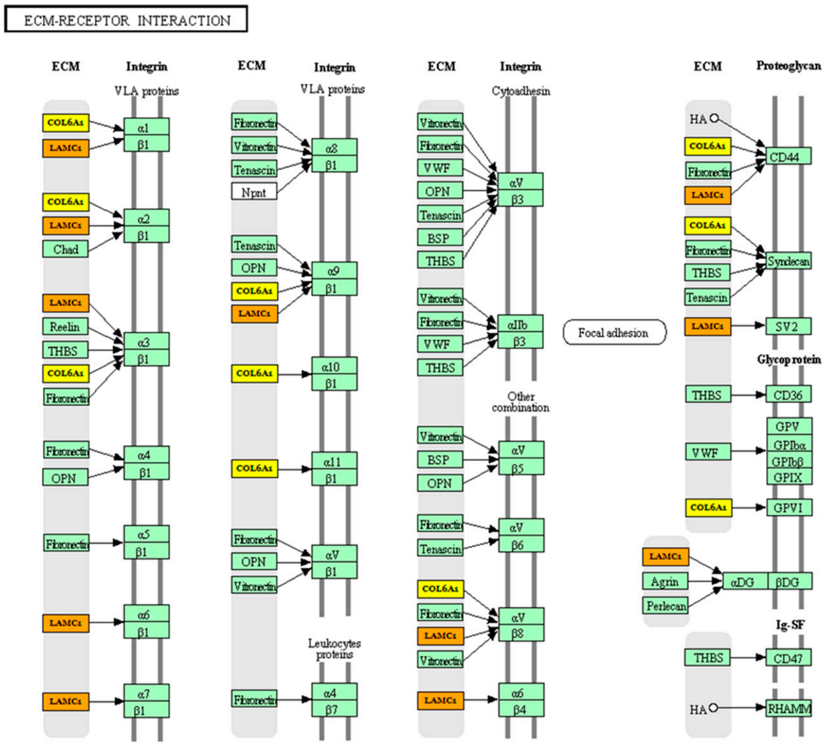

Figure S2 MiR-28-5p and miR-708 regulate ECM-Receptor interaction genes. MiR-28-5p and miR-708-5p regulate ECM-Receptor interaction genes. (A) ECM-receptor interaction genes, yellow represents genes targeted by a single transcript, and orange represents genes regulated by both transcripts. The red circles highlight genes correlated with OS in NSCLC

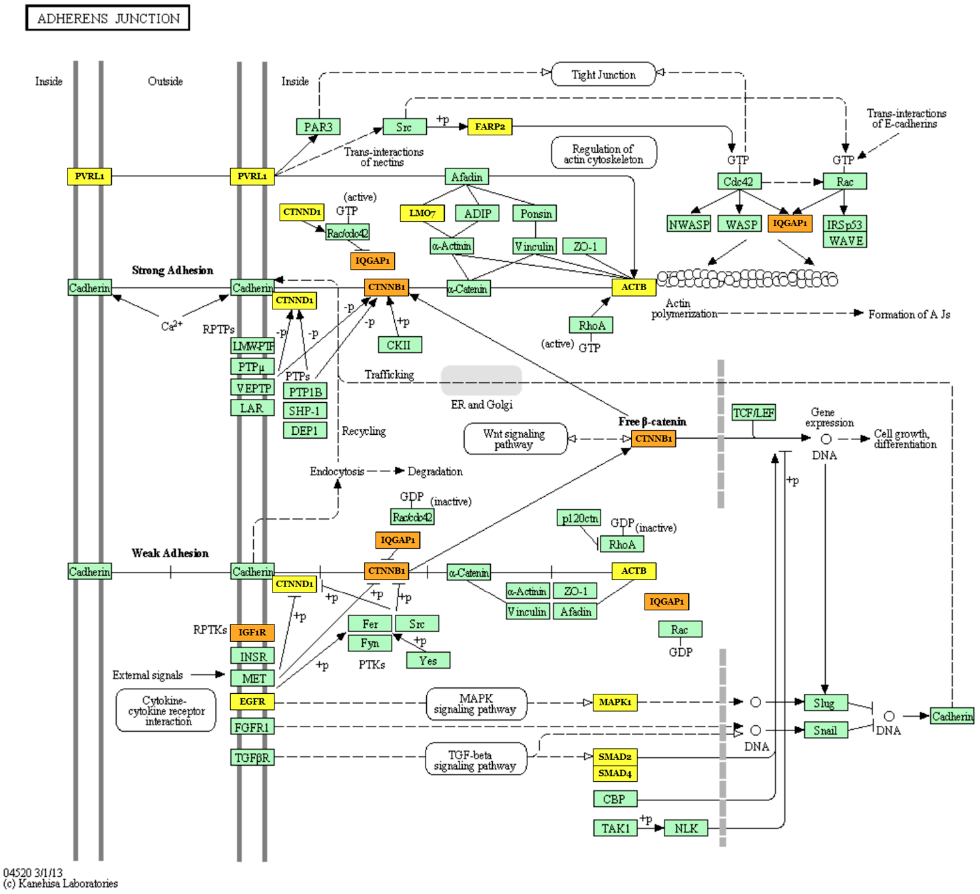

Figure S3. MiR-28-5p and miR-708 regulate the adherents junction' genes. Yellow represents genes targeted by a single transcript, and both transcripts regulate genes marked in orange. The red circles highlight genes correlated with OS in NSCLC.

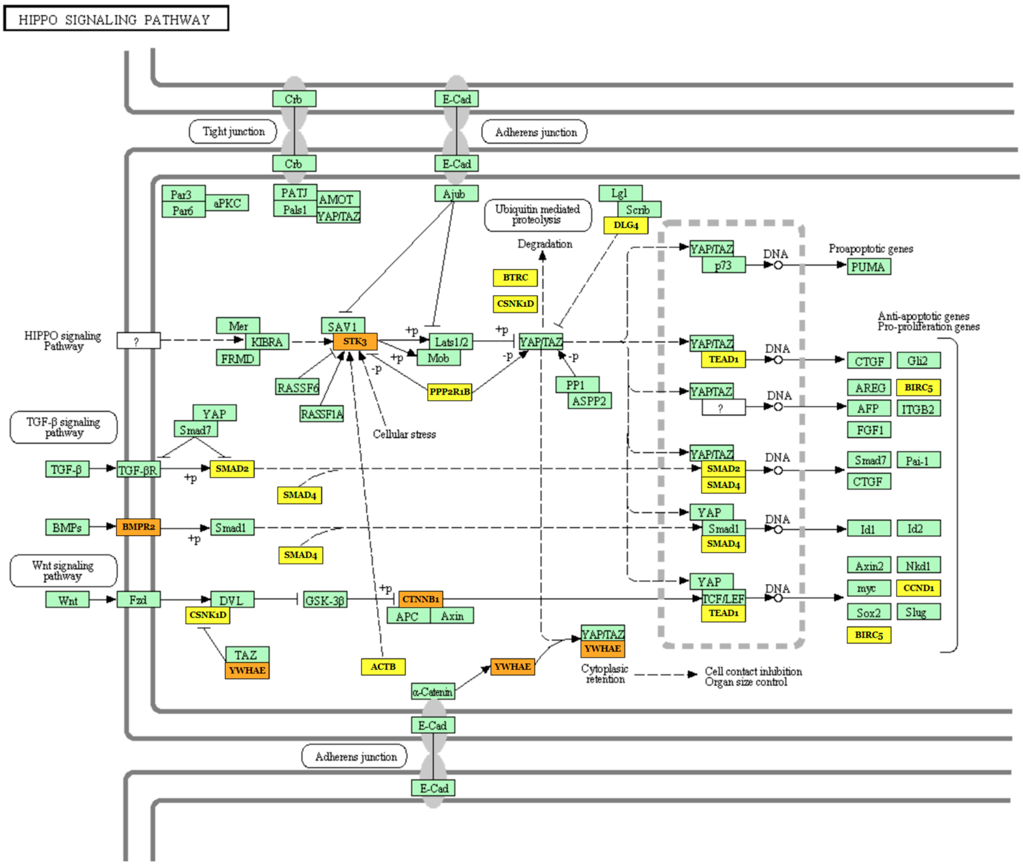

Figure S4. MiR-28-5p and miR-708 regulate the Hippo signaling-related genes. Yellow represents genes targeted by a single transcript, and both transcripts regulate orange genes.
